# Univariate and multivariate sex differences and similarities in gray matter volume within essential language-processing areas

**DOI:** 10.1186/s13293-023-00575-y

**Published:** 2023-12-21

**Authors:** Carla Sanchis-Segura, Rand R. Wilcox, Alvaro Javier Cruz-Gómez, Sonia Félix-Esbrí, Alba Sebastián-Tirado, Cristina Forn

**Affiliations:** 1 Departament de Psicologia Bàsica Clinica I Psicobiología, Facultat de Ciències de La Salut, Universitat Jaume I, Avda Sos Baynat, SN, 12071 Castelló, Spain; 2 Department of Psychology, University of Southern California, Los Angeles, USA; 3 Departamento de Psicología, Universidad de Cádiz, Cádiz, Spain

**Keywords:** Sex differences, Sex similarities, Gray matter volume, Language, Multivariate

## Abstract

**Background:**

Sex differences in language-related abilities have been reported. It is generally assumed that these differences stem from a different organization of language in the brains of females and males. However, research in this area has been relatively scarce, methodologically heterogeneous and has yielded conflicting results.

**Methods:**

Univariate and multivariate sex differences and similarities in gray matter volume (GM_VOL_) within 18 essential language-processing brain areas were assessed in a sex-balanced sample (N = 588) of right-handed young adults. Univariate analyses involved location, spread, and shape comparisons of the females’ and males’ distributions and were conducted with several robust statistical methods able to quantify the size of sex differences and similarities in a complementary way. Multivariate sex differences and similarities were estimated by the same methods in the continuous scores provided by two distinct multivariate procedures (logistic regression and a multivariate analog of the Wilcoxon–Mann–Whitney test). Additional analyses were addressed to compare the outcomes of these two multivariate analytical strategies and described their structure (that is, the relative contribution of each brain area to the multivariate effects).

**Results:**

When not adjusted for total intracranial volume (TIV) variation, “large” univariate sex differences (males > females) were found in all 18 brain areas considered. In contrast, “small” differences (females > males) in just two of these brain areas were found when controlling for TIV. The two multivariate methods tested provided very similar results. Multivariate sex differences surpassed univariate differences, yielding "large" differences indicative of larger volumes in males when calculated from raw GM_VOL_ estimates. Conversely, when calculated from TIV-adjusted GM_VOL_, multivariate differences were "medium" and indicative of larger volumes in females. Despite their distinct size and direction, multivariate sex differences in raw and TIV-adjusted GM_VOL_ shared a similar structure and allowed us to identify the components of the SENT_CORE network which more likely contribute to the observed effects.

**Conclusions:**

Our results confirm and extend previous findings about univariate sex differences in language-processing areas, offering unprecedented evidence at the multivariate level. We also observed that the size and direction of these differences vary quite substantially depending on whether they are estimated from raw or TIV-adjusted GM_VOL_ measurements.

**Supplementary Information:**

The online version contains supplementary material available at 10.1186/s13293-023-00575-y.

## Plain language summary

While it is generally assumed that there is a distinct organization of language in the brains of females and males, studies investigating potential sex-based differences in language-related neural circuits have been characterized by their methodological heterogeneity and yielded inconclusive results. In this study, we explored how the brains of men and women differ in a well-defined network of brain areas essential for basic language functions. We found that there are indeed differences in the size of certain brain regions involved in language, with men and women showing varying patterns of these differences. Interestingly, the way these differences were observed depended on whether they are assessed at the whole network or at individual brain regions. Also, when considering the size of these brain regions in relation to overall cranial volume, the differences changed. So, this study highlights that understanding these brain differences requires considering different factors, like existing sex differences in cranial size, and looking at local effects but also their interactions and relationships in the broader context of functional brain networks.

**Supplementary Information:**

The online version contains supplementary material available at 10.1186/s13293-023-00575-y.

## Highlights

- There are consistent gray matter volume (GM_VOL_) differences between males and females in brain regions associated with language processing.
- Multivariate analyses at the whole network level reveal larger sex differences in GM_VOL_ compared to univariate comparisons at individual brain regions.
- The direction and magnitude of these univariate and multivariate differences vary significantly depending on whether raw or TIV-adjusted GM_VOL_ is used, emphasizing the importance of considering TIV-related variations.
- The structure of multivariate sex differences is quite similar when inferred from raw and from TIV-adjusted GM_VOL_, hence allowing the identification of the brain areas that more significantly contribute to the neuroanatomical divergences of this network in females and males.

## Introduction

Language is fundamental of nearly every facet of human cognition and behavior. It is also frequently cited as a domain in which sex differences have been established (e.g., [1–4]) and commonly affected in developmental, psychiatric, and neurological diseases with a sex-biased prevalence and/or prognosis (e.g., [5–7]). While it is generally assumed that these behavioral and clinical differences arise from a distinct organization of language in the brains of females and males, studies investigating potential sex-based anatomical and functional differences in language-related neural circuits are surprisingly limited [3, 8] and have yielded inconclusive results [2, 3, 8, 9]. Thus, for example, there are only two studies that have explored possible sex differences in gray matter volume (GM_VOL_) at the inferior frontal gyrus (IFG) in children, and they reported contradictory sex effects (Blanton et al., males > females [10]; Wilke et al., females > males [11]). These and similar conflicting findings might lead to the interpretation that there are no sex differences in the IFG and in other language-processing brain areas. However, this lack of consistency in the results of the studies assessing possible sex differences in brain structure and function related to language can, at least in part, be attributed to methodological heterogeneity observed across these studies [3, 8, 12].

One factor that likely affects the replicability in these studies is the lack of consensus regarding which brain areas can be considered “language areas” and how exactly they should be anatomically parcellated and labeled [13, 14]. In this regard, it should be noted that many language studies have focused on lateralization and have reported sex differences in some brain structures located in the right hemisphere. However, these brain regions do not appear to be “essential language areas” but rather responsible of other processes, such as selective attention, context/prosody processing, and manipulation verbal material in working memory, which are recruited during specific tasks or functions but not others [13, 15–17]. Consequently, the involvement of these structures and the effects of sex that may be observed in them may exhibit lower replicability across studies [15, 17, 18]. On the other hand, classical descriptors for essential language-processing brain regions in the left hemisphere, such as “Broca’s area” or “Wernicke’s area”, have been used so liberally that they no longer have clear anatomical meanings, to the point that some researchers have proposed discontinuing their use [14, 19, 20]. This ambiguity also extends to other anatomical descriptors related to language, which could help explain the low replicability of sex effects in these brain regions. For example, as mentioned earlier, Blanton et al. [10] and Wilke et al. [11] found opposite sex differences in the left IFG, but they also defined this region differently. While Blanton et al. [10] pre-defined the IFG as a single region of interest (ROI) that included its orbitalis, opercularis, and triangularis subregions, Wilke et al. [11] employed voxel-based analyses without predefining any ROI. This makes unclear to what extent both studies were examining a comparable anatomical region despite employing the same anatomical label. Another example of how the definition of ROIs can affect the identification of sex differences in language-processing areas, even when evaluated in a single sample, was provided by Harrington et al. [21]. This neurofunctional study did not identify any effect of sex in the activation of the IFG when this anatomical region was defined as a single ROI but found opposite sex effects in the same participants when separately assessing task-induced activations in the three IFG subdivisions [21]. Therefore, as these examples illustrate, what may appear to be contradictory results when analyzing the effects of sex in a particular anatomical region may actually be different effects in different anatomical regions conflated under the same anatomical label, just as similar findings may be reported under different anatomical descriptors.

While the parcellation of ROIs is a crucial aspect, it is not the only variable that differs and that has probably contributed to disparate findings in this research area. For instance, the studies of Blanton et al. [10] and Wilke et al. [11] also diverged in terms of sample size, statistical significance thresholds, and the inclusion of covariates of no interest in their analyses. The interplay of sample size (often small in such studies [2, 8]) and significance thresholds (which have not always been corrected for multiple comparisons [12]) is of paramount importance because it essentially determines the likelihood of finding a statistically significant effect as well as their reliability/ reproducibility [22, 23]. Furthermore, although it is known that language abilities and its neural underpinnings can be affected by variables such as age, handedness, educational and socio-economic level [12, 24–26], not all studies have statistically controlled for all these covariates. Similarly, the impact of male–female differences in total intracranial/ brain volume (TIV/TBV) is of major importance when exploring volumetric sex differences, but only some studies have taken into account and, among those that did, some (e.g., [11, 27–29]) have used simple proportions or other methods that may introduce biases leading to larger relative gray matter (GM) volumes in females [30–33].

**Fig. 1.**
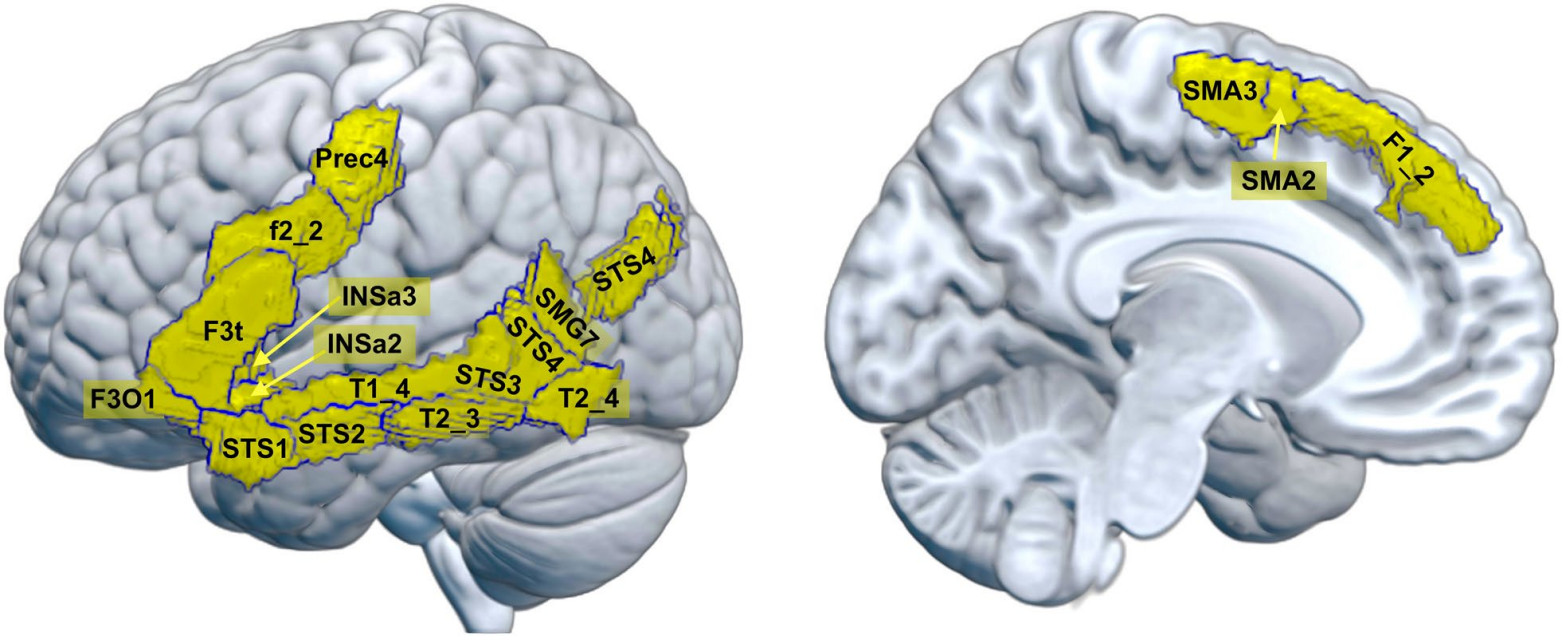
The SENT_CORE network. The SENT_CORE network (in yellow) is the larger of the three language-related networks included in the SENSAAS atlas developed by Labache et al. [13], and it includes 18 of its 32 regions. The SENT_CORE network (also referred as the LANG network) comprises the cortical areas essential for sentence processing and which lesion causes aphasia. The full names corresponding to the abbreviations used in this figure plus additional information are provided in Table 1

The present study was designed to address these limitations and describe the sex similarities and differences in GM_VOL_ at the brain areas subserving basic language functions. Specifically, we utilized a large (*n* = 588) sample of right-handed young adults with no significant differences in age, handedness scores, overall cognitive status, educational or socio-economic levels. We conducted our assessments using both raw GM_VOL_ estimates and after adjusting these estimates with the well-validated [30, 31, 33] power-corrected proportions method [34]. Furthermore, our ROIs were selected, parcellated, and labeled according to the only available anatomical atlas specifically constructed to include essential language areas and networks in right-handed healthy individuals (the SENSAAS atlas; [13]). Within this atlas, we focused on the so-called SENT_CORE network (see Table 1 and Fig. 1), consisting of 18 ROIs of the left hemisphere that have been consistently reported in meta-analyses of healthy individuals mapped during language tasks [20, 35] and that lead to aphasia when damaged [13, 36]. Lastly, given that these areas form a coherent functional network, we conducted both univariate and multivariate analyses using robust statistical methods capable of providing meaningful effect sizes for individual network components as well as the entire network [37].

**Table 1:** The SENT_CORE network

ROI name (AICHA atlas)	Abbreviation	*X* (mm)	*Y* (mm)	*Z* (mm)	Associated terms
S_Precentral-4	prec4	− 42.2	0.7	49.9	Sentence (9.01), comprehension (8.12), language (7.65)
G_Frontal_Sup-2	F1_2	− 11.9	46.5	41.4	Inferences (5.82), medial prefrontal (5.62), social (4.88)
S_Inf_Frontal-2	f2_2	− 43.1	14.8	29.4	Semantic (10.85), phonological (9.21), lexical (6.76), sentence (5.96)
G_Frontal_Inf_Tri-1	F3t	− 49.4	25.6	4.7	Semantic (12.31), sentences (10.89), words (9.76)
G_Frontal_Inf_Orb-1	F3O1	− 42.2	30.5	− 16.9	Semantic (9.19), words (6.86)
G_Insula-anterior-2	INSa2	− 33.8	16.8	− 12.7	Olfactory (7.93), amygdala/hippocampus (5.99), salience network (4.17)
G_Insula-anterior-3	INSa3	− 33.7	23.7	0.6	Gain (10.18), difficulty (6.44), orthographic (4.48)
G_Temporal_Sup-4	T1_4	− 58.7	− 23.3	3.7	Auditory (22.09), speech (16.77), listening (16.06)
G_Temporal_Mid-3	T2_3	− 61.0	− 35.0	− 4.8	Comprehension (8.64), sentence (7.98), semantic (5.7), syntactic (5.14)
G_Temporal_Mid-4	T2_4	− 53.1	− 59.4	7	Temporal (7.24), Temporal sulcus (7.09), language (4.21)
S_Sup_Temporal-1	STS1	− 49.7	14	− 21.5	Comprehension (9.03), sentences (8.26), semantic (7.92), syntactic (4.25)
S_Sup_Temporal-2	STS2	− 54.9	− 7.2	− 12.8	Sentences (9.85), comprehension (8.87), semantic (7.46), syntactic (5.89)
S_Sup_Temporal-3	STS3	− 54.7	− 33.0	− 1.7	Sentences (10.95), language (10.03), comprehension (8.57), syntactic (6.28)
S_Sup_Temporal-4	STS4	− 56.5	− 48.4	13.4	Sentences (10.21), comprehension (8.53), speech (8.22)
G_SupraMarginal-7	SMG7	− 55.2	− 51.7	25.5	Theory mind (6.7), mind (6.1), mind tom (5.49)
G_Angular-2	AG2	− 37.5	− 70.4	39.5	Retrieval (9.03), semantic (6.29)
G_Supp_Motor_Area-2	SMA2	− 10.6	18.2	63.1	Personal (5.84), autobiographical (4.86), mentalizing (3.91)
G_Supp_Motor_Area-3	SMA3	− 7.2	7.6	65.6	Reappraisal (6.22), comprehension (6.1), language (5.5)

The table displays the ROI names, abbreviations, and MNI space coordinates of the mass center of 18 local volumes of the SENT_CORE network of the SENSAAS atlas. The last column contains some of the terms more tightly associated to the anatomical coordinates of each of these areas according to Neurosynth as well as the z-score value obtained in the meta-analytical association test. Note that ROIs are named and abbreviated as in the AICHA atlas because this is the atlas that was used to construct the SENSAAS atlas, although, as mentioned in the main text, there is a lack of consensus in the labeling of language-processing areas and some these ROIs can be found under other names (e.g., *F2_2* is named F3opd in [35] and mFG in [20]; see [13] for details)

## Materials and methods

### Participants

The present study was conducted using data from the 1,200 Subject Release of the Human Connectome Project (HCP), which includes structural magnetic resonance imaging (MRI) data from 1,113 healthy young adult participants (606 females and 507 males) [38]. From this dataset, a final sex-balanced sample of 588 right-handed participants (294 females, 294 males) was extracted using the following procedure: (1) because the SENSAAS atlas has been constructed for right-handed individuals, participants with scores ≥ 50 in the Edinburgh Handedness Inventory were preselected (*N* = 931, 512 females, 410 males). (2) From this pool of subjects, a maximum of two individuals per family was randomly selected (*N* = 741, 413 females, 328 males). (3) Eleven and five individuals were excluded because of missing values in scores of total cognition adjusted by age (CogTotalComp_AgeAdj) and socio-economic level (SSAGA_income), respectively. (4) On the remaining subjects, the downSample function of the caret package for R [39] was used to randomly select the same number of females and males so that both classes had the same frequency as the minority class in each age range defined in the variable "Age range" of the HCP dataset (specifically, 56 individuals of 22–25 years, 144 individuals of 26–30 years, 92 individuals of 31–35 years, and 2 individuals over 36 years).

As indicated by their medians and inter-quartile ranges (IQR), the 294 female and 294 male participants finally selected had similar ages (medians = 29.05 and 28.45 years, *p* = 0.118; IQR = 6 and 4), handedness (medians = 83.06 and 86.11, *p* = 0.175; IQR = 15 and 16), overall cognition (medians = 113.72 and 115.25, *p* = 0.441; IQR = 27.97 and 28.57), educational level (medians = 16 and 15.99, *p* = 0.288; IQR = 2 and 2), and socio-economic level (as derived from reported income; medians = 5.88 and 5.79, *p* = 0.755; IQR = 2 and 2). Moreover, none of these variables showed a statistically significant correlation with the raw or TIV-corrected volumes of the 18 brain regions defined in the SENT_CORE network of the SENSAAS atlas (Additional file 1: Table S1A-2). Therefore, it might be reasonably assumed that the sex differences and similarities estimated in the present study were not affected by these potential confounders.

On the other hand, the selected sample included 24 and 25 pairs of monozygotic and dizygotic twins (8.16% and 8.5% of the selected sample), proportions that are substantially lower than those observed in the original HCP dataset (24.21% and 26.78%, respectively).

### Imaging and data preprocessing

#### MRI acquisition and images preprocessing

The MRI acquisition details for the HCP-sample can be found in the reference manual of the S1200 release of the HCP (https://www.humanconnectome.org/storage/app/media/documentation/s1200/HCP_S1200_Release_Reference_Manual.pdf).

Brain images were preprocessed with the CAT12.2 toolbox (http://www.neuro.uni-jena.de/cat/, version r1290) of the SPM12 (http://www.fil.ion.ucl.ac.uk/spm/software/spm12/, version 7219) software. CAT12 preprocessing was conducted according to the standard default procedure suggested in the manual (https://neuro-jena.github.io/cat12-help/). Briefly, it includes the following steps: (1) segmentation of the images into gray matter, white matter, and cerebrospinal fluid; (2) registration to a standard template provided by the International Consortium of Brain Mapping (ICBM); (3) DARTEL normalization of the gray matter segments to the MNI template; (4) modulation of the normalized data via the “affine + non-linear” algorithm; and (5) data quality check (in which no outliers or incorrectly aligned cases were detected). Images were not smoothed because we were only interested in the modulated images.

After applying this procedure, which does not include any correction for overall head size, voxels were mapped into the 18 regions of the SENT_CORE network of the SENSAAS atlas (see Fig. 1 and Table 1; for a complete description, see [13]) by calculating the total gray matter volume for each region of interest (ROI) and participant via a MATLAB script (https://www0.cs.ucl.ac.uk/staff/g.ridgway/vbm/get_totals.m). TIV was estimated using native-space tissue maps obtained in the segmentation step. More specifically, TIV was calculated as the sum of gray matter, white matter, and cerebrospinal fluid total values multiplied by voxel size and divided by 1,000 to obtain a milliliter (ml) measurement.

#### TIV adjustment: the raw and the PCP datasets

Previous studies have shown that the estimates of univariate and multivariate sex differences are largely dependent on TIV variation, but also that not all the currently used methods are equally effective and valid for removing TIV-variation [30, 31, 40]. Therefore, in the present study, all analyses were conducted twice on the same subjects, without introducing any TIV adjustment (“raw” dataset) and after removing TIV variation with the well-validated [34] *power-corrected proportions* (PCP) method (PCP dataset). The PCP method improves the traditional proportions approach by introducing an exponential correcting parameter in the denominator. Thus the adjusted volume for a specific brain region is calculated as VOL_adj_ = VOL/TIV^b^, where the b parameter corresponds to the slope value of the LOG(VOL) ~ LOG(TIV) regression line calculated for the entire sample of participants [34].

To enhance comparability and avoid possible distortions due to their different ranges [41, 42], the volumetric scores of the raw and the PCP datasets were transformed to robust z-scores, using the formula: robust z-score = 0.6745(xi–median)/median absolute deviation. This formula includes the appropriate rescaling coefficient (0.6745), so the median absolute deviation is put in the same scale than the normal standard deviation, so the obtained scores can be numerically interpreted as conventional z-scores [43].

### Statistical analyses

All statistical analyses were conducted based on the raw and the PCP datasets using different packages for R [44]. Except noted otherwise, p-values were adjusted for multiple comparisons using the FDR-method [45], and only effects remaining statistically significant after this correction are discussed in the main text (although unadjusted and adjusted p-values for all effects are provided in Additional file 1). In addition to statistical significance, descriptive methods and measures of effect size were used that are based on robust, non-parametric techniques that do not assume normality nor homoscedasticity [12]. The functions used are described in [12] and included in the Rallfun-v41 file, which is freely accessible at https://osf.io/xhe8u/.

#### Univariate analyses

To offer a complete perspective of the univariate differences and similarities between females and males in the raw and in the PCP datasets, five complementary strategies were used. More specifically:

1. Differences between the females’ and males’ distributions of the robust z-scores corresponding to each of the 18 brain areas included in the SENT_CORE network were globally estimated using a heteroscedastic analog of the Wilcoxon–Mann–Whitney (WMW) test.
2. The overall degree of similarity between these distributions was quantified using the *η* overlap index, which measures the area intersected by two probability density functions but, conversely to other overlap measures, $\widehat{\eta }$(the sample estimate of *η*) can be calculated in the absence of symmetry, unimodality, or any other distributional assumption [46]. In the present study, kernel density estimation (KDE) and $\widehat{\eta }$ were obtained through the *boot.overlap* (1000 repetitions) function of the *overlapping* package for R [47].
3. The decile values of these distributions were compared with the *qcomhd* function (see [37]), which uses the Harrell–Davis quantile estimator in conjunction with a percentile bootstrap approach (4,000 repetitions) to calculate the deciles, their between-groups differences, and their 95% confidence intervals (CI) while adjusting the significance level for multiple comparisons. Because these calculations were conducted on robust z-scores, the obtained differences between deciles (hereinafter denoted as $\widehat{d}$) provide a standardized effect size index which can be numerically interpreted in the same way that Cohen’s d values. Therefore, the size of these differences were qualified as "large", "medium", "small", or "negligible" according to the benchmarks commonly used to characterize Cohen's d values [48]
4. The *cidv2* function (see [12]) was employed to calculate the probability of superiority (PS). The PS is defined as P(A > B), the probability that a randomly sampled member of group A will have a higher score than the score attained by a randomly sampled member of group B [49]. A 95% confidence interval for Cliff’s delta value [50], which is P(A > B)-P(A < B), was computed and its statistical significance was tested. The size of the obtained Cliff’s delta values were qualified as "large", "medium", "small", or "negligible" according to the benchmarks provided in [51]. Moreover, to add further perspective, we also calculated Cohen’s U3 [48, 49], another dominance effect size index that quantifies the percent of cases of group A that have scores larger than the median score of group B. Although this index is often derived from a formula assuming normality [48, 52], in the present study U3 was empirically calculated by directly counting the cases of group A with larger scores than the median score of group B and their non-parametric 95% CI were estimated through the bootstrap percentile interval (2,000 repeats) with the *boot.ci* function of the *boot* package [53].
5. Possible sex differences in scatter, skewness, and kurtosis were also assessed. Differences in scatter were evaluated with the function *IQR2g.W* that employs the Harrell–Davis quantile estimator in conjunction with a Wald-type test to compare the inter-quartile ratios of two groups (see [37]). Differences in skewness and kurtosis were assessed with the function *pb2gen* that uses a percentile bootstrap method (2000 repeats) to estimate the statistical significance of the difference between any estimates of two independent samples [37].

Previous studies have shown that estimates of raw GM_VOL_ are directly related to TIV [34, 54, 55] and that the strength of these relationships (slope values and/ or R^2^ of linear TIV-VOL_raw_ regressions) is correlated with the size of the sex differences found in these VOL_raw_ [30, 31]. Conversely, VOL_adj_ with appropriate methods no longer show any relationship with TIV, and the size of the sex differences in GM_VOL_ are uncorrelated with the TIV-VOL_adj_ regression parameters [30, 31, 34]. Therefore, in the present study, we employed Spearman’s rho to evaluate the extent the univariate sex differences observed in the raw and in the PCP datasets were related to the amount of variance explained by TIV (R^2^) in each VOL_raw_/VOL_adj_ of these datasets.

#### Multivariate analyses

As initially proposed by Lippa and Connelly [56], we used classification probabilities as a continuous dependent variable on which individual and between-group multivariate similarities and differences can be quantified with a variety of effect size indexes (for a recent example, see [40]). More specifically, in the present study, we utilized the *lrm*, *validate, calibrate* and *pentrace* functions of the *rms* package [57] to implement, bootstrap-validate, and calibrate two L2-penalized logistic regression (LR) models. These LR models aimed to predict sex categories from the 18 regional brain volumes included in the SENT_CORE network in the raw and in the PCP datasets, respectively. The sex category used as reference in each of these two LR models was chosen according to the direction of the observed univariate differences of the corresponding dataset, so higher Pclass scores were associated with larger raw or TIV-adjusted GM_vol_.

From the fitted models, optimism-corrected models’ discrimination indexes (Nagelkerke’s R2, Somers’ Dxy, the C-index) as well as individual classification probabilities (hereinafter referred to as Pclass scores) were obtained. The univariate vector of Pclass scores was analyzed using the tests and metrics described in Sect. 2.3.1. However, in this case, depictions of the males and females’ cumulative density functions (CDFs) were also included, making it possible to visually illustrate how males and females compare between them in three complementary ways: 1) the proportion of cases in each group with Pclass scores equal to or lower/ higher than any possible cutoff can be directly estimated (e.g., 0.5 to estimate the achieved accuracy, an effect size typically employed in classification studies); 2) the proportion of individuals of one group that have Pclass values equal to or lower than a given proportion of individuals of the other group (e.g., the median to obtain the Cohen’s U3 value); and, 3) by comparing the Pclass values at specific quantiles (the decile values) of the females/ males’ distributions. In addition, because it offers a complementary perspective to that offered by the males-females comparisons previously described (see [58]), the distribution of all pairwise differences between females and males was calculated and described in terms of its deciles and the percent of differences indicating larger GM_VOL_ in the reference sex category in each dataset. Of note, because Pclass scores range between 0 and 1 the size of the group differences calculated on this dependent variable can be gauged in terms of their percent to the maximum possible (POMP scores, [59]).

The results of the analyses conducted with Pclass scores were validated by comparing them to those obtained with an alternative method with different mathematical foundations. More specifically, multivariate sex differences and similarities were re-assessed using the multivariate projection-type analogue of the Wilcoxon–Mann–Whitney test implemented by the *mulwmwv2* function [37]. To our knowledge, this method has not been previously employed to assess multivariate brain differences between females and males, but, as described in [60], this method is specifically designed to compare two groups in terms of P(A < B) on a projection of the multivariate data. Thus, in contrast with most classic multivariate statistics (e.g., Hotelling’s *T*^2^) and with classification-based procedures, it does not incorporate any arbitrariness about the direction of the observed between-groups differences. Moreover, the *mulwmwv2* function does not only provide a PS estimate (and its 95% CI that allows testing the null hypothesis of *P*(*X* < *Y*) = 0.5 at *p* < 0.05), it also makes it possible to obtain and R^2^ effect size and the individual distances to the multivariate center which can be used as individual continuous scores to assess within- and between-sex similarities and differences with other statistical methods. Therefore, in the present study, some of the tests and metrics described in Sect. 2.3.1 (overlap, PS, Cliff’s delta, and Cohen’s U3) were applied to these distance-based scores and the obtained results were compared to those gathered when using Pclass scores. In addition, we estimated Q [61], a classification-based effect size similar to the accuracy obtained from the LR models but that can be calculated from any continuous measurement and that allowed an additional comparison of the magnitude of the multivariate sex differences calculated from the distance-based and the Pclass scores. Finally, the possible relationships between these distances and TIV values and Pclass scores were evaluated using the *rhohc4bt* function [37], which estimates Pearson’s correlations using a bootstrap-t method in conjunction with the HC4 estimator.

Finally, the architecture of the fitted LR models was explored by building up their corresponding nomograms and by correlational analyses involving the LR coefficients. Nomograms are graphical calculating devices that consist of a series of scales (one by each variable included in the model) whose length is proportional to its relative importance to the final model. Although nomograms are ordinarily used to predict individual classification probabilities, in this case they were used to describe how the females’ and males’ scores in these variables added up into a final composite from which Pclass scores are non-linearly derived. To assess whether Pclass scores adequately recapitulate the identified univariate effects, the relationship between the coefficient values in these models and the size of the univariate differences (as estimated from the medians’ differences, the Cliff’s delta, and the Cohen’s U3 values) in each predictor in the raw and in the PCP dataset was assessed with the Spearman’s rho correlation index. The absolute value of the Spearman’s rho correlation index (denoted as ($\left|rho\right|$) was also employed to quantify whether the regression coefficients in these two LR models had a similar ordering (that is, to assess till which extent the same brain features contributed the most in both models). These nomograms were also compared to others obtained after fitting LR models that incorporated TIV as an additional predictor. This last comparison served to compare the relative importance of TIV to that of the rest of predictors, and also provided an unprecedented experimental confirmation of the adequacy of the PCP method when aiming to statistically control the influence of TIV-variation in multivariate scenarios.

## Results

### Univariate sex differences and similarities

**Fig. 2.**
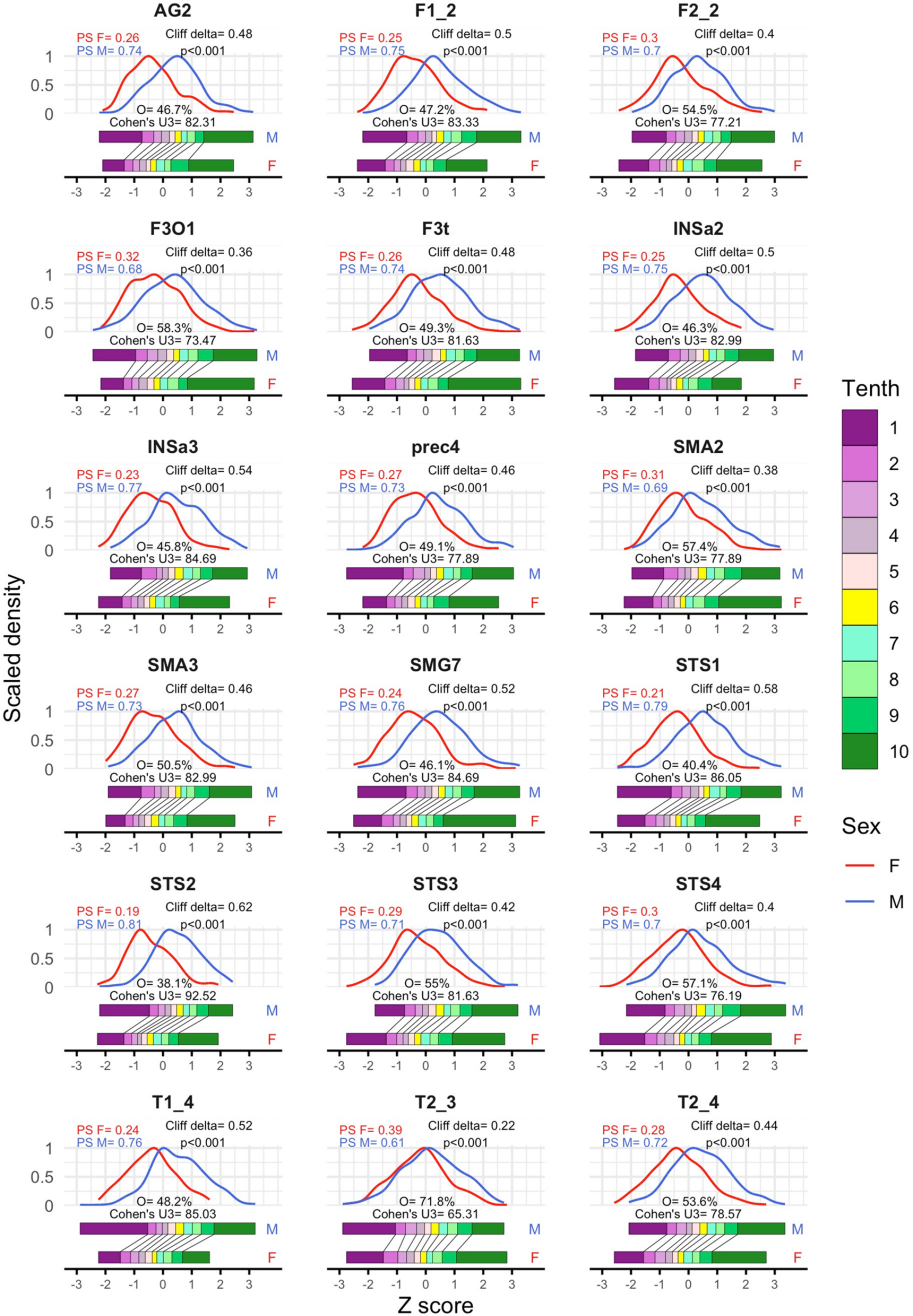
Univariate sex differences and similarities in the raw dataset. Panels depict the distributions of females (red) and males (blue) z-scores and report the percent of mutual overlap and the estimated Cohen’s U3 values in each of the 18 brain areas included in the SENT_CORE network. Under these density-based depictions: the tenths (deciles) of the same distributions of males (top) and females (bottom) are displayed as colored rectangles, whereas black solid segments are used to denote statistically significant differences in the values of the deciles that define these tenths. At the top of the panels, the probability of superiority of males (blue) and females (red) and the corresponding Cliff’s delta statistic are reported (including the associated p-value only in those cases in which it remained statistically significant after multiple comparisons correction) (M = males, F = females, PS = probability of superiority, O = overlap)

Males exhibited larger raw GM_vol_ than females in the 18 areas comprised in the SENT_CORE network (Fig. 2). Thus, the males’ distributions of the robust z-scores of these raw volumes were significantly shifted towards higher values (median shift = 0.78z, *p* < 0.001 in all cases; Additional file 1: Table S1B), so in all these brain areas the majority of males had GM_vol_ scores that were larger than the females’ median (Cohen’s U3 range = 65.31–92.52%) and the males and females’ distributions exhibited generally “low” levels of overlap (median = 49.21%; see Fig. 2 and Additional file 1: Table S1C). Moreover, statistically significant and, in most cases, “large” M > F differences were found at all decile values of these 18 distributions (range $\widehat{d}$= 0.27–1.20, median $\widehat{d}$= 0.80, *p* < 0.01 in all cases; Fig. 2 and Additional file 1: Table S1D). Accordingly, the probability that a randomly picked male would exhibit a larger GM_vol_ score than a female (PS-M) was found to be larger than its counterpart (PS-F) in each and every brain area, hence resulting in Cliff’s delta values spanning between 0.22 (*T2_3*) and 0.62 (*STS2*) with a median value of 0.47 (*p* < 0.001 in all cases; Fig. 2 and Additional file 1: Table S1E). In contrast, across all the brain areas considered, females and males seemed to exhibit a similar shape and spread as no statistically significant differences in their respective skewness, kurtosis, or IQR estimates were found (Additional file 1: Table S1F).

Taken together, these results indicate that males and females exhibit widespread and “large” differences in their respective amounts of raw GM_vol_ in the 18 areas of the SENT_CORE network. However, as summarized in Table 2, the sizes of the observed sex differences in local GM_vol_ were highly correlated with the variance accounted for by TIV in each of these 18 brain areas (see R^2^ and other regression outputs in Additional file 1: Table S1G), hence indicating that differences in raw GM_vol_ are largely dependent on gross morphology differences between females and males.

**Table 2:** Influence of TIV in the observed differences and similarities in the raw and in the PCP datasets

Raw dataset				PCP-dataset			
Statistic	rho	p value	fdr- *p* value	Statistic	rho	*p* value	fdr- *p* value
Quantile				Quantile			
0.1	0.511	0.003	0.004	0.1	0.273	0.024	0.221
0.2	0.589	*p* < 0.001	0.002	0.2	0.049	0.126	0.283
0.3	0.612	*p* < 0.001	0.002	0.3	0.046	0.173	0.283
0.4	0.631	0.001	0.002	0.4	0.073	0.189	0.283
0.5	0.616	*p* < 0.001	0.002	0.5	0.030	0.352	0.352
0.6	0.653	*p* < 0.001	0.002	0.6	0.036	0.344	0.352
0.7	0.593	0.002	0.003	0.7	0.009	0.334	0.352
0.8	0.573	0.004	0.005	0.8	0.123	0.094	0.282
0.9	0.120	0.120	0.120	0.9	0.337	0.071	0.282
Cliff’s delta	0.697	0.001	0.001	Cliff’s delta	− 0.001	0.996	0.996
Cohen’s U3	0.671	0.002	0.002	Cohen’s U3	− 0.053	0.836	0.836
Overlap	-0.686	0.002	0.002	Overlap	− 0.179	0.478	0.478

Spearman’s rho correlations were calculated between the proportion of variance explained by TIV (R^2^) and the estimates of the sex differences/similarities (differences at each decile value, Cliff’s delta, Cohen’s U3, and percent of overlap) obtained in the raw and in the PCP dataset

**Fig. 3:**
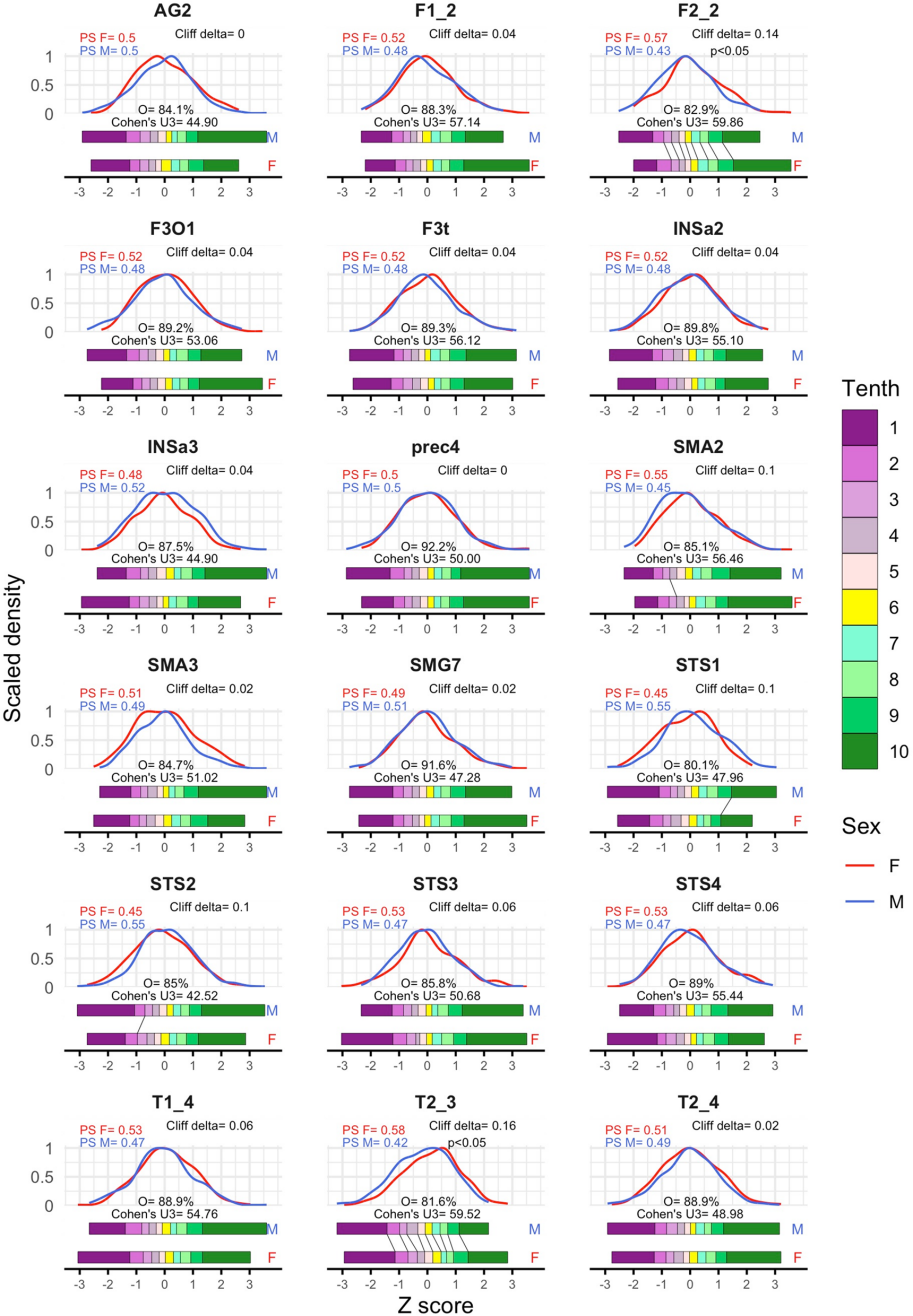
Univariate sex differences and similarities in the PCP dataset. Panels depict the distributions of females (red) and males (blue) z-scores and report the percent of mutual overlap and the estimated Cohen’s U3 values in each of the 18 brain areas included in the SENT_CORE network. Under these density-based depictions, the tenths of the same distributions of males (top) and females (bottom) are displayed as colored rectangles, whereas black solid segments are used to denote statistically significant differences in the values of their deciles. At the top of the panels, the probability of superiority of males (blue) and females (red) and the corresponding Cliff’s delta statistic are reported (including the associated p-value in those cases in which it remained statistically significant after multiple comparisons correction) (M = males, F = females, PS = probability of superiority, O = overlap)

Controlling for TIV-related variation resulted in a suppression of most, but not all, of the previously observed sex differences in local GM_vol_. Thus, in the PCP dataset (Fig. 3), the robust z-scores’ distributions of females and males were very much alike and WMW tests revealed statistically significant differences after p-values correction in just 2 brain areas (*T2_3* and *F2_2*), in both of which females exhibited slightly larger scores than males (estimated shifts: 0.28 and 0. 25z, respectively; *p* < 0.05 in both cases; Additional file 1: Table S2B). Accordingly, Cohen’s U3 values were close to the 50% in all brain regions and all the females and males’ distributions exhibited a “large” degree of mutual overlap (median = 87.42%, see Fig. 3 and Additional file 1: Table S2C). As a result, few statistically significant differences were found when comparing the deciles of these distributions (Fig. 3, Additional file 1: Table S2D). These differences were again “small” in size and their direction varied for different brain areas. Thus, F > M differences were predominant and specifically found in all deciles’ values of *T2_3*, in the D2-D9 values of *F2_2*, and the D3 value of *SMA2* (range $\widehat{d}$= − 0.2, − 0.39; median = − 0.28), whereas M > F differences where only found for the D9 ($\widehat{d}$= 0.39) and the D2 ($\widehat{d}$= 0.27) values of *STS1* and *STS2*, respectively (Fig. 3, Additional file 1: Table S2D). Accordingly, in most areas the probability that a randomly picked female (PS-F) would exhibit a larger GM_vol_ score than a male was similar to its counterpart PS-M in most brain areas, and, once again, only in *T2_3* and *F2_2* the difference between PS-M and PS-F achieved statistical significance (Cliff’s delta = 0.16 and 0.14, respectively; *p* < 0.05; Fig. 3, Additional file 1: Table S2E). Finally, no statistically significant sex differences in shape (skewness and kurtosis) nor spread were observed in any brain region (Additional file 1: Table S2F).

Taken together these results suggest that, when the contribution of gross morphology differences between females and males to local brain volumes is ruled out, females and males are very similar regarding their GM_vol_ in the majority of the brain regions of the SENT_CORE network. Thus, only in two brain areas (*T2_3* and *F2_2*) “small” but consistent F > M differences were confirmed through distinct statistical approaches. As could be expected, the size of these differences was uncorrelated with TIV (Table 2) and TIV did not explain any variance in these brain sites (Additional file 1: Table S2E).

### Multivariate sex differences and similarities

#### Estimating multivariate sex differences and similarities from classification probabilities

To evaluate the possible multivariate sex differences and similarities in the SENT_CORE network as a whole in the raw and PCP datasets, the information of its 18 brain regional components was condensed into a unidimensional metric space defined by the classification probabilities (Pclass scores) provided by two independent logistic regression models. The reference category of each model was chosen according to the direction of the observed univariate differences of each dataset, so higher Pclass scores were associated with larger amounts of raw or TIV-adjusted GM_vol_, respectively (see details in Sect. 2.3.2). The fitted LR models identified a statistically significant relationship between the predictors and sex categories in both the raw (*χ*^2^_(16.36)_ = 324.34, *p* < 0.001) and the PCP (*χ*^2^_(14.12)_ = 59.48, *p* < 0.001) datasets. The discrimination indexes associated with these LR models indicated that multivariate sex differences would probably be “large” in the raw dataset (*R*^2^ = 0.55, C = 0.89, Dxy = 0.79) but “small” in the PCP dataset (*R*^2^ = 0.11, C = 0.68, Dxy = 0.37). This initial inference was confirmed by all subsequent analyses.

**Fig. 4:**
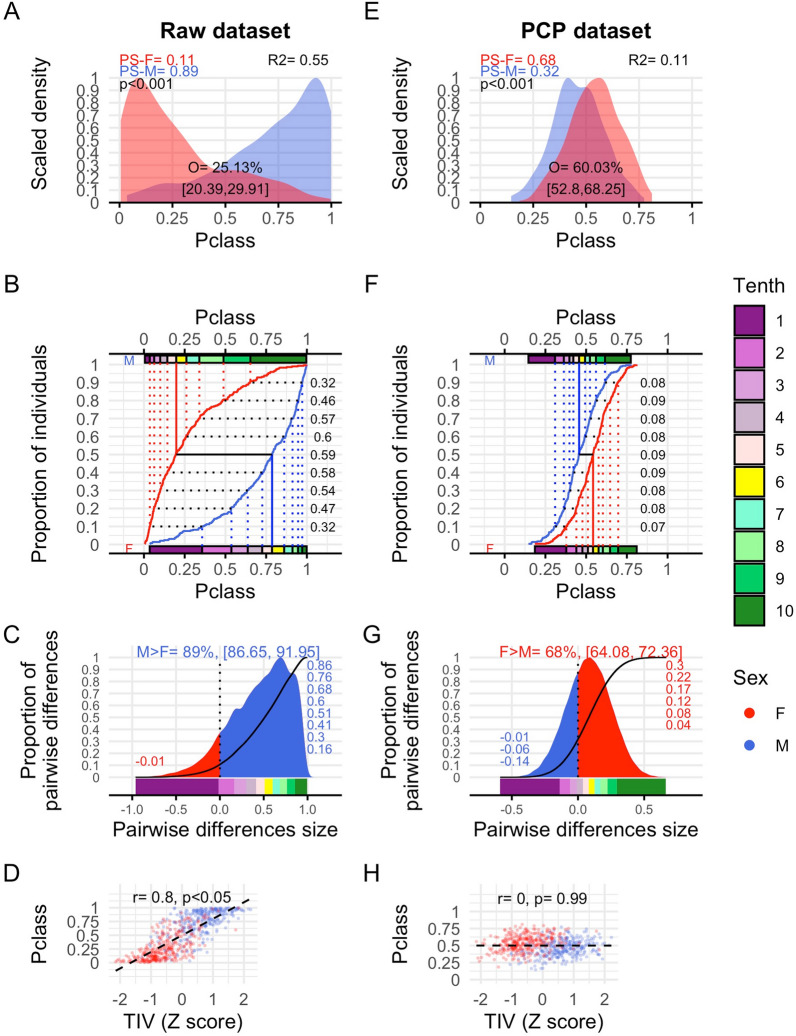
Multivariate sex differences and similarities estimated from Pclass scores in the raw and PCP datasets. **A** and **E** Scaled density function for the males (blue) and females’ (red) distributions of the Pclass scores in the raw and PCP datasets. Within each panel, the percent of overlap (O) as well as the probabilities of superiority for males and females (PS) as well as the p-value associated and an *R*^2^ effect size index derived from the comparison of these PS values using Cliff’s delta are provided. Panels **B** and **F** Cumulative density functions (CDF) of Pclass scores of males (blue) and females (red), along with the tenths of these distributions (colored rectangles), the decile values (vertical lines). The size of the estimated sex differences in these deciles is also included (see further details of these comparisons in Additional file 1: Table S3C). **C** and **G** Scaled density functions of all the pairwise differences between females and males. Within each density plot, two different areas are colored according to the direction of these differences (F > M, red; M > F, blue) and, at the top of the panel, the percent of pairwise differences favoring the reference sex category (males in the raw dataset, females in the PCP dataset) as well as its 95% CI are included. Each of these two panels also includes the CDFs of pairwise differences’ distributions (black line), the tenths of these distributions (colored rectangles) and the size of the estimated deciles’ differences. **D** and **H** Scatterplots depicting the bivariate relationship (quantified by a robust analog of Pearson’s r correlation index) between the Pclass and TIV scores (M = males, F = females)

Figure 4A displays the distributions of the males and females’ pclass-scores obtained when using the raw amounts of GM_vol_. Both males and females exhibited highly skewed and opposing distributions (skewness_M_ = − 0.99, skewness_F_ = 1.01, *p* < 0.001), with most of the females accumulating near the lower bound of the Pclass continuum, and most of the males accumulating near the upper bound. These distributions did not seem to differ in kurtosis (kurtosis_M_ = 3.17, kurtosis_F_ = 3.10, *p* = 0.905) or spread (IQR_M_ = 0.35, IQR_F_ = 0.32, *p* = 0.417) but they clearly did in location, hence exhibiting a “small” degree of mutual overlap (25.13%). Thus, the probability that a randomly chosen male would have a Pclass score higher than that of randomly chosen female was “large” (PS-M = 0.89) and significantly higher than its counterpart (PS-F = 0.11; Cliff’s delta = 0.78, *p* < 0.001; Additional file 1: Table S3A).

In contrast, when Pclass scores were calculated from TIV-adjusted GM_vol_ estimates (PCP dataset; Fig. 4E), the males and females’ Pclass-scores were much more symmetrically distributed (skewness_M_ = − 0.16, skewness_F_ = 0.16, *p* = 0.298), and both of them occupied the most central regions of the Pclass continuum without apparent differences in kurtosis (kurtosis_M_ = 2.77, kurtosis_F_ = 2.61, *p* = 0.508), or spread (IQR_M_ = 0.16, IQR_F_ = 0.16, *p* = 0.744) and showing just slight differences in location. Consequently, both distributions exhibited a substantial degree of overlap (60.03%) between them, and the PS of the reference sex category (in this case, the females) was significantly different but not much larger than that of the alternative sex category (PS-F = 0.68, PS-M = 0.32; Cliff’s delta = 0.36, *p* < 0.001; Additional file 1: Table S3A).

To delve deeper into the characterization of these multivariate sex differences, Fig. 4B displays the empirical cumulative distribution functions (CDFs) of the males’ and females’ Pclass-scores obtained in the raw dataset, along with their respective decile values. This figure makes it possible to compare females and males in three complementary ways (see Sect. 2.3.2), leading to the following main observations: 1) 80.61% of females and 82.93% of males were below and above the 0.5 cutoff value classically used in classification studies, then resulting in a classification accuracy of 81.77%; 2) 96.26% of males had Pclass scores that were higher than or equal to the median Pclass score of the females (Cohen’s U3; Additional file 1: Table S3B); 3) Statistically significant M > F differences were found at all decile values (range $\widehat{d}$= 0.32–0.6, *p* < 0.001 in all cases; Additional file 1: Table S3C). Accordingly, when all M-F pairwise differences were calculated (Fig. 4C), M > F differences were far more frequent (89%) and expectable (86–92%) than F > M differences and, in the majority of the cases, these observed differences were “moderate” to “large” in size (e.g., 50% of the differences had a size equal of larger than 50% of the maximum possible). However, individual Pclass scores were highly correlated to TIV (rho = 0.80, *p* < 0.001; Fig. 4D), hence suggesting that sex differences estimated from these Pclass scores could be largely driven by the differences between males and females in TIV values.

In contrast, as shown in panels F-G of Fig. 4, the same comparisons indicated that when TIV-related variation was statistically controlled (see Panel H of the same figure), multivariate sex differences were much smaller. More specifically: (1) the percent of correctly classified cases dropped to 63.09%; 2) Cohen’s U3 and Cliff delta values were substantially smaller than those observed in the raw dataset (75.51% *vs.* 96.26% and 0.36 *vs.* 0.78, respectively; Additional file 1: Table S3A, B); (3) although statistically significant F > M differences were observed in all decile values of the Pclass distribution, the size of these differences” ranged between 0.07 and 0.09 (*p* < 0.001 in all cases; Additional file 1: Table S3C). As a result, the distribution of all pairwise differences between females and males was quite symmetrical and centered close to the zero value (0.08), hence indicating that F > M differences were just slightly more frequent (68%) and expectable (64–72%) than M > F differences. Moreover, most of the observed pairwise differences were “small” in size (e.g., 80% of the observed differences had a size that was equal to or less than 20% of the maximum possible).

Taken together, these results indicate that, when the SENT_CORE network is taken as a whole, males have larger amounts of raw GM_vol_ than females but also that, as already observed when the 18 brain areas composing this network were analyzed separately, these volumetric measures (and, therefore, their mutual differences) are largely driven by the existing M > F differences in TIV. In fact, when statistically controlling for TIV-variation, larger relative amounts of GM_vol_ in females should be expected although the observed multivariate differences should be much smaller.

#### Validating and interpreting the multivariate sex differences and similarities estimated from classification probabilities

**Fig. 5:**
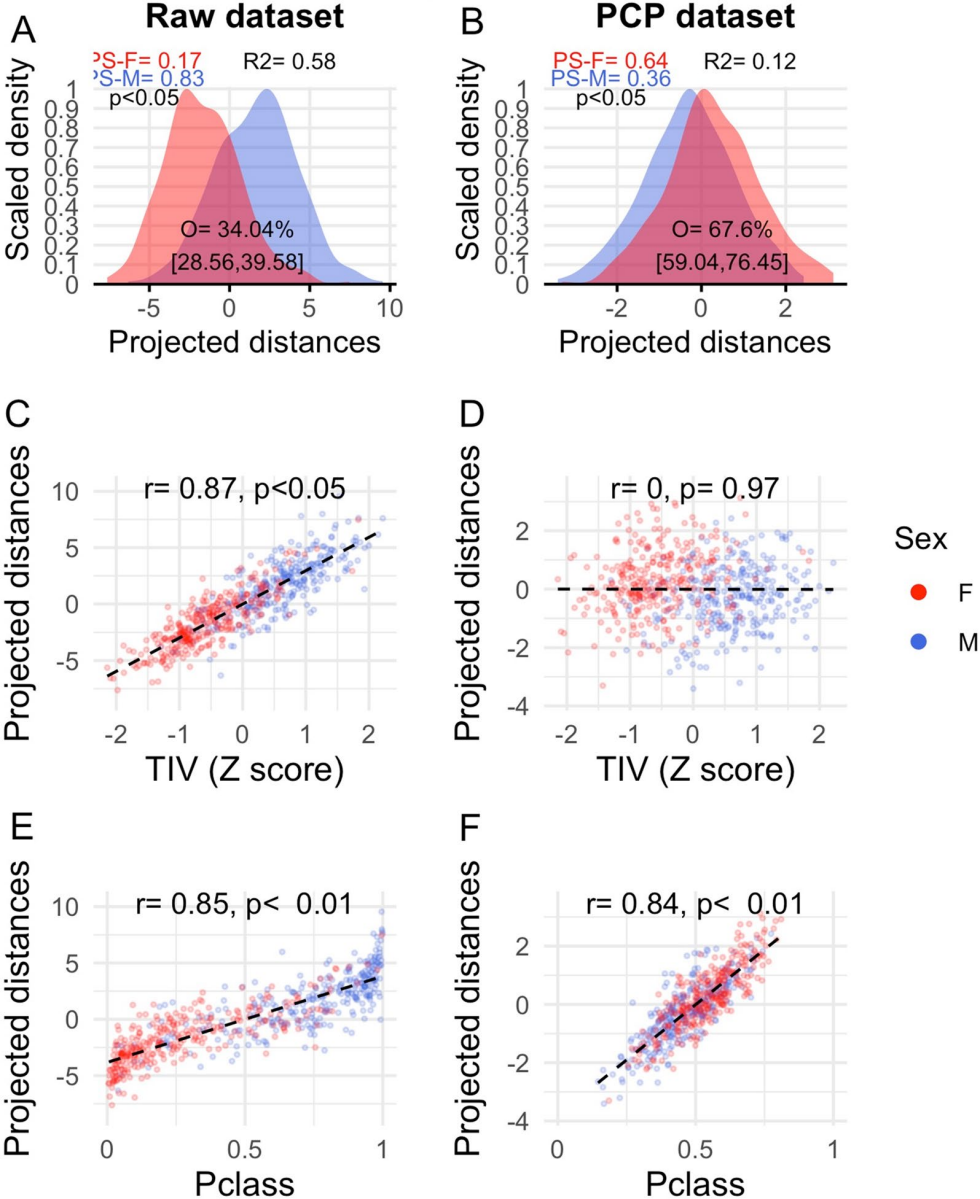
Multivariate sex differences and similarities estimated from projected distances in the raw and PCP datasets. **A** and **E** Scaled density function for the males (blue) and females’ (red) distributions of the projected distances’ scores in the raw and PCP datasets. Within each panel, the percent of overlap (O) as well as the probabilities of superiority for males and females (PS) as well as the p-value associated and an *R*^2^ effect size index derived from the comparison of these PS values using Cliff’s delta are provided. **C** and **D** Scatterplots of the bivariate relationships (quantified by a robust analog of the correlation coefficient Pearson’s *r*) between the projected distances and TIV scores in the raw and PCP datasets. **E** and **F** Scatterplots of the bivariate relationships (quantified by a robust analog of the correlation coefficient Pearson’s *r*) between the projected distances and Pclass scores in the raw and PCP datasets (M = males, F = females)

To validate and gain additional insight on the multivariate sex differences in the SENT_CORE network estimated from Pclass scores, additional analyses were conducted. Firstly, multivariate sex differences and similarities were re-assessed using a very different statistical approach sustained on a projection pursuit method that unambiguously assesses the direction of these differences and allow estimating their size with the same indexes used for Pclass scores (see details in Sect. 2.3.2 and [60]). The results of this re-assessment are summarized in Fig. 5 and, as it can be readily observed, they confirmed the correctness of the direction imposed to those obtained from Pclass scores and provided very similar estimates in terms of size (see Table 3). Moreover, individual scores based on projected distances calculated from raw, but not from PCP-adjusted, GMvol showed a similar dependency on TIV values than Pclass scores (*r* = 0.87, *p* < 0.01 and *r* < 0.01, *p* > 0.970, respectively; Fig. 5C, D). Additionally, in both the raw and the PCP datasets, individual projected distances were significantly correlated with individual Pclass scores (*r* = 0.85, *p* < 0.01 and *r* = 0.84, *p* < 0.01, respectively; Fig. 5E, F). Taken together, these results suggest that, despite their different mathematical foundations, Pclass scores and projected distances capture the same multivariate reality and provide nearly identical estimates of the multivariate sex differences and similarities in the SENT_CORE network.

**Table 3:** Comparison of the multivariate sex differences estimated from Pclass scores and projected distances

	Raw dataset		PCP dataset	
	Projected distances	Pclass	Projected distances	Pclass
PS-M	0.83 [0.80, 0.86]	0.89 [0.86, 0.92]	0.36 [0.31, 0.4]	0.32 [0.28, 0.36]
PS-F	0.17 [0.14, 0.2]	0.11 [0.08, 0.14]	0.64 [0.6, 0.69]	0.68 [0.64, 0.72]
Cliff’s delta	0.66 [0.6, 0.73]	0.78 [0.73, 0.83]	0.28 [0.2, 0.37]	0.36 [0.28, 0.45]
Overlap	34.04% [28.56, 39.58]	25.13% [20.39, 29.91]	67.6% [59.04, 76.45]	60.13% [52.8, 68.25]
Cohen’s U3	90.48% [86.73, 95.24]	96.26% [92.18, 98.3]	72.45% [64.29, 77.89]	75.51% [67.35, 81.97]
Wilcox and Muska’s Q	0.75	0.81	0.60	0.63
R^2^	0.58	0.55	0.12	0.11

The table shows the similarity of the point estimates of the probability of superiority (PS) for females and males, the associated Cliff’s delta value, the percent of overlap between the females and males’ distributions, the Cohen’s U3, the Wilcox and Muska’s Q, and R^2^ statistics obtained from Pclass scores and projected distances. The 95% CI of these estimates are provided between square brackets

**Fig. 6.**
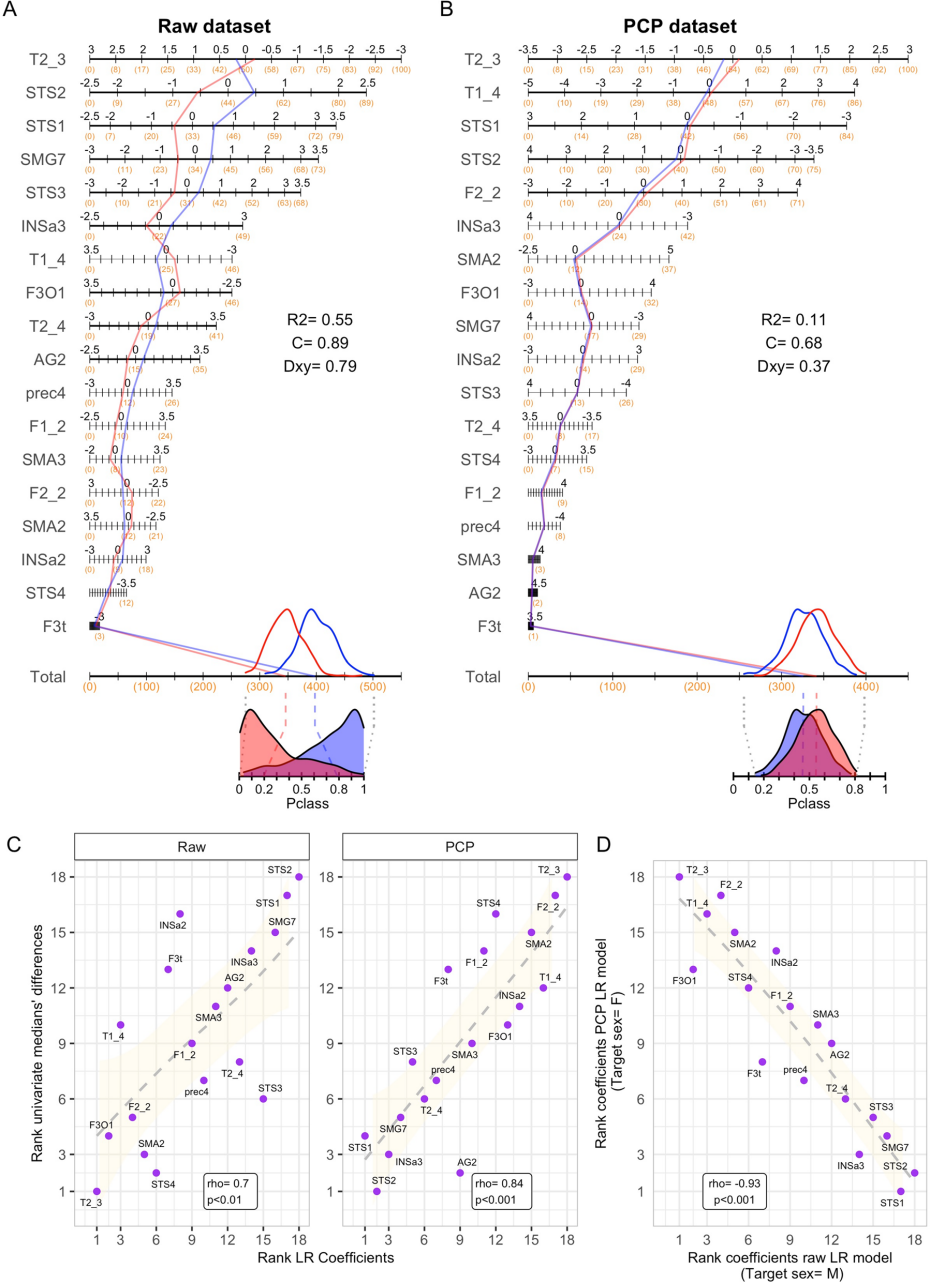
Structure of the LR models fitted in the raw and PCP datasets. **A** and **B** Nomograms illustrating the relative contribution of each component to the SENT_CORE network to the Pclass scores yielded by the LR models fitted in the raw (reference sex category: males) and in the PCP (reference sex category: females) datasets. The values of three discrimination indexes (*R*^2^, C index, and Somers’ D) of each of these two models are reported within the plots. Although nomograms are ordinarily used to predict individual classification probabilities, in this case, the males (blue) and females (red) medians are used to represent how the scores of these groups in each feature were scored (i.e., points; orange numbers), additively integrated in composites (“total points”), and non-linearly project to the Pclass continuum on which the multivariate sex differences and similarities displayed in Fig. 4 were estimated. Note that to enhance readability: (1) brain features are decreasingly sorted according to their contribution to the model; (2) instead of including a points’ axis, the points achievable (orange numbers) in each scale are represented back-to-back to the features’ values (black numbers); (3) the marks of some scales have been suppressed; and, (4) to highlight them, the scales of those features achieving statistical significance are depicted with thicker lines. **C** Ordinal relationship (quantified through the Spearman’s rho correlation index) between the regression coefficient values and the size of the univariate differences (medians’ difference) in the raw (left) and PCP (right) datasets. Note that the sign of this association is largely arbitrary as it arises from the different sex category used as reference in the raw and PCP models. **D** Ordinal relationship (quantified through the Spearman’s rho correlation index) between the coefficient values of the LR models fitted in the raw and PCP datasets. To ease the visualization of the relationships depicted in panels **C** and **D**, trend lines obtained through gam-smoothing (and their 95% interval; yellow shade) have been added

Secondly, to obtain further insight on the structure of the multivariate sex differences estimated from Pclass scores, the nomograms of the LR models fitted in the raw and PCP datasets were built up (Fig. 6A, B). These nomograms illustrate the relative contribution of each brain area to the final model (length and maximum of points assigned to each scale), but also how the scores of males and females (as represented by their medians) in each of these weighted dimensions were additively integrated into overall scores that non-linearly project into the Pclass continuum. Of note, the relative importance of these 18 brain areas for the final LR models (quantified in terms of the regression coefficient values) exhibited a similar ordering than that of their univariate sex differences (assessed in terms of medians’ difference, Cliff’s delta, or Cohen’s U3), hence resulting in direct and statistically significant correlations between both sets of observations (rho = 0.7, 0.67, 0.68 and rho = 0.84, 0.86, 0.82 in the raw and in the PCP dataset, respectively; see Fig. 6C and Additional file 1: Fig. S1).

**Fig. 7.**
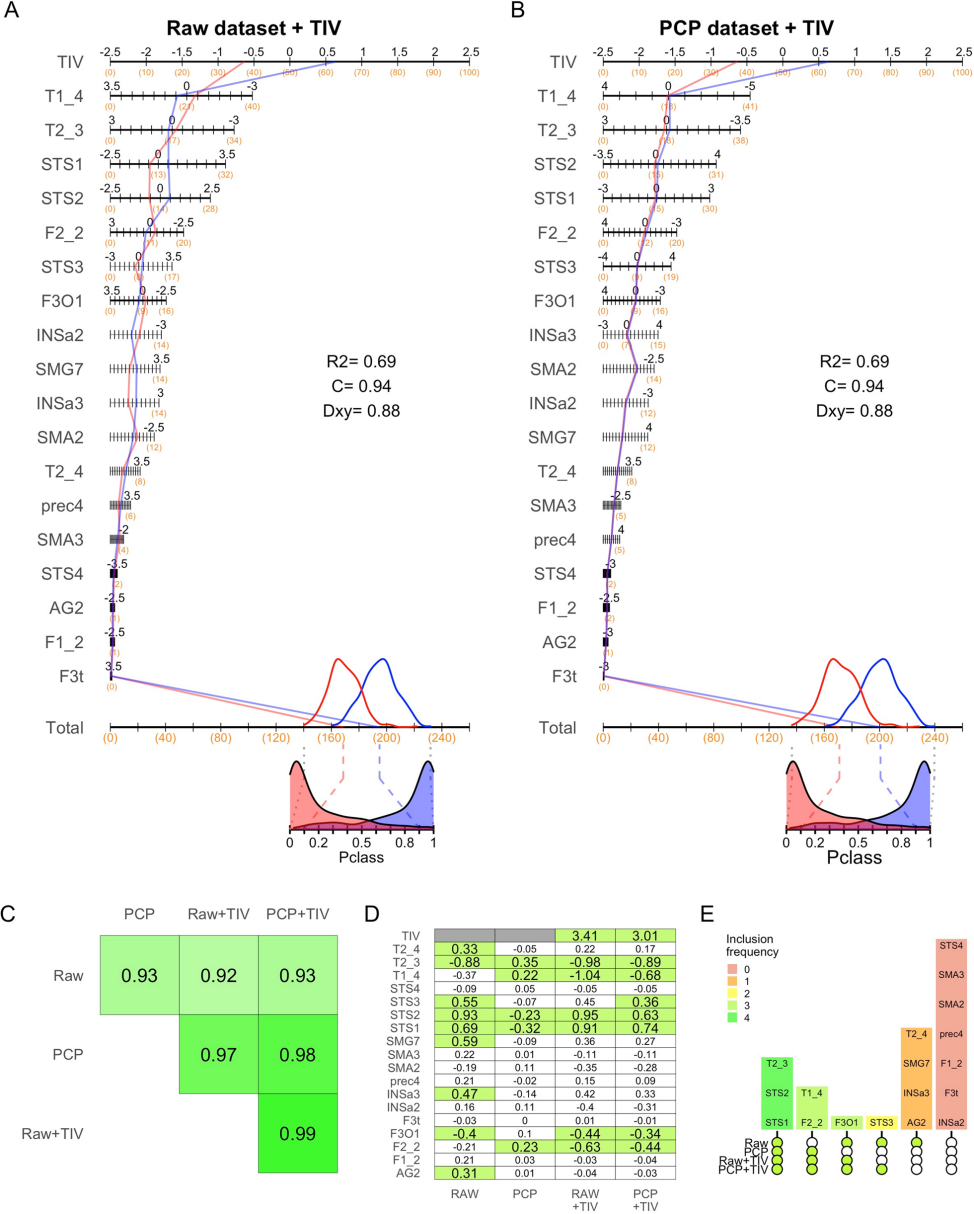
Structure of the LR models after including TIV as an additional predictor. **A**, **B** Nomograms illustrating the relative contribution of TIV and each of the features included in the raw and PCP datasets to the Pclass scores. The values of three discrimination indexes (*R*^2^, C index, and Somers’ D) of each of these two models are reported within the plots. **C** Ordinal relationships (quantified through the absolute value Spearman’s rho correlation index) between the coefficient values of the four LR models fitted in this study and depicted in **A** and **B** of Figs. 6 and 7). Note that these associations were calculated excluding the coefficient value associated to TIV (which is only included in two of these four LR models) and that, because the sign of these associations is arbitrary (i.e., it arises from the different sex category used as reference in the distinct LR models), absolute rho values are reported. **D** Values of the regression coefficients in each of the four LR models fitted in the present study. Highlighted in green are those coefficients reaching statistical significance (*p* < 0.05) in each model (see details in Additional file 1: Table S3D). **E** UpSet plot illustrating the intersections between the predictors reaching statistical significance in the fitted LR models. In this plot: (1) the color of the line-joined circles denotes whether the features listed in each column reached statistical significance (green) or not (white) in a particular model, thus identifying which models are part of each intersection; (2) the height of the bars of bars on the top illustrates the number of features included in each intersection (the cardinality of each intersection); (3) the color of the bars denotes the number of models that included the listed features as significant predictors. Thus, for example, the first intersection includes three brain features that reached statistical significance as predictors in all four models, the second one includes two brain features that reached statistical significance in the PCP, raw + TIV, and PCP + TIV models (but not in the raw model), and so on

Figure 6 also allows noticing that, although the multivariate sex differences observed in the raw dataset are much larger than those observed in the PCP dataset, the nomograms obtained in these two datasets were remarkably similar (although the direction of their axes are reversed due to the use of different sex categories as reference in the LR models). Indeed, the values of the regression coefficients of these two LR models exhibited a very similar ordering ($\left|rho\right|$= 0.93, *p* < 0.001; panel D). This suggest that statistically controlling TIV-variation affects the size of the multivariate sex differences in GM_VOL_ at the SENT_CORE network, but it does not artefactually alter their structure. This conclusion was confirmed after rebuilding the same models but adding TIV as an additional predictor. Thus, as shown in panels A and B of Fig. 7, the nomograms obtained were virtually identical and their regression coefficients showed an almost perfect correlation between them ($\left|rho\right|$>0.99, *p* < 0.001). Furthermore, the ordering of the regression coefficients corresponding to the components of the SENT_CORE network in these two last LR models was also very similar to that observed in the other two previously fitted LR models ($\left|rho\right|$> 0.92 in all cases; Fig. 7C).

Finally, given the structural similarities of these four LR models, we conducted an assessment across models aimed to identify which components of the SENT_CORE networks more consistently contributed to the multivariate effects observed in this network. From the nomograms of these models (and even better so from the heatmap panel D of Fig. 7), it can also be readily observed that three brain features achieved statistical significance as predictors in all LR models, whereas others solely achieved significance in some of them, and yet other seven features did not exhibit a significant predictive value in any model. Thus, after conducting an intersection analysis of the coefficients reaching statistical significance in these four LR models (Fig. 7E), it can be suggested that *T2_3, STS1, STS2* but also *F2_2, T1_4,* and *F3O1* were the areas of the SENT_CORE network that more consistently contribute to the multivariate separation between males and females when considering this network as a whole.

## Discussion

As described in the introduction section, there have been relatively few studies dedicated to examining potential neuroanatomical differences between females and males in brain areas responsible for essential language functions and the findings from these studies have yielded inconclusive results. The current study contributes to this research area: (1) by extending the current knowledge about the univariate within- and between-sexes similarities and differences in GM_VOL_ at language-processing areas; (2) by introducing, comparing, and validating statistical methods to assess these similarities and differences at the multivariate level; and, (3) by providing unprecedented estimates of these multivariate differences.

### Univariate sex differences and similarities in language-processing brain areas

In line with previous studies, our research aimed to describe univariate sex differences and similarities in GMVOL within various language-processing areas. However, our analysis incorporated three distinctive and unprecedented characteristics: (1) targeted brain areas—we focused our evaluation in all the brain areas comprising the SENT_CORE network of the SENSAAS atlas, which is specifically designed for the assessment of high-order sentence processing in healthy individuals [13]; (2) robust statistical methods—unlike most prior studies that primarily relied on parametric between-mean comparisons without reporting effect sizes, we employed several robust, non-parametric tests and effect size indexes that do not assume normality nor homoscedasticity and allow us to explore the entire female and male distributions, quantifying their similarities and differences in location, spread, and shape; (3) raw and TIV-adjusted estimates—our assessment was conducted in parallel with raw and TIV-adjusted estimates of GM_VOL_ (raw and PCP datasets), explicitly examining the influence of this covariate.

Our findings revealed that in both the raw and PCP datasets, within every brain region considered, males and females exhibited similar distribution shapes without significant differences in skewness, kurtosis, or spread (see Figs. 2 and 3). In contrast, consistent and statistically significant differences in location were observed between these male and female distributions. Notably, the number, size, and direction of these sex effects varied considerably depending on whether raw or TIV-adjusted GM_VOL_ estimates were compared. Thus, in the raw dataset, statistically significant differences (males > females) were evident in all brain regions. These differences were robust, consistent (i.e., found with several statistical procedures and at different location measures) and, according to widely used benchmarks for various effect size indexes [48, 51], “large” in all regions except for the *T2_3* area, where sex effects appeared “small” (Fig. 2 and Additional file 1: Table S1D, F). In contrast, the PCP dataset revealed consistent and robust sex differences in only two brain areas, *T2_3* and *F2_2.* Interestingly, these differences indicated larger amounts of TIV-adjusted GM_VOL_ in females, and their magnitude was “small” (Fig. 3 and Additional file 1: Table S2D, F).

Given that this study is the first to assess sex differences in the SENT_CORE language network identified by Labache et al. [13], direct comparisons with previous research are challenging. Nonetheless, our findings appear to align with some prior observations. Specifically, our results are in agreement with those of other large-sample studies that have consistently reported “large” univariate sex differences indicating larger raw GM_vol_ in males across nearly all brain regions but less abundant and “small” sex differences with site-specific direction when comparing TIV-adjusted GM_vol_ estimates (e.g., [30, 31, 33, 62, 63]). Furthermore, our observation that females exhibited greater TIV-adjusted GM_vol_ in the *T2_3* and *F2_2* regions seems consistent with previous studies reporting larger relative TIV-adjusted GM_vol_ in women in similar or overlapping stereotactic coordinates and anatomical descriptors within the temporal (e.g., [64–66]) and inferior frontal (e.g., [65, 67]) cortices. However, these results should be regarded with caution until replicated by other studies employing the brain parcellation proposed by the SENSAAS atlas.

### Multivariate sex differences: why and how

In part due to the limitations in traditional multivariate methods used for group comparisons (e.g., MANOVA [68, 69], neuroimaging studies—including those aimed to describe sex differences in language-processing areas—have been often relied on univariate comparisons, which separately assess the potential effects of sex in each brain region [70, 71]. However, cognitive/behavioral processes as language do not emerge from the activity of isolated brain regions but from the coordinated operations of localized but distributed neuronal networks, and, within these networks, the relevance of each neural component depends on its interaction with the rest of components [71, 72]. Therefore, univariate comparisons have limited value as they only reveal potential group differences at the level of individual network components but do not provide insights into differences that may exist at the whole network level [72–75].

To address this limitation, some recent studies have adopted the analytical strategy initially suggested by Lippa and Connelly [56]. They use classification probabilities (here referred to as Pclass) generated by regression/ classification methods as a continuum that condenses information from multiple brain features and that enables the estimation of multivariate sex differences (e.g., [40, 76, 77]). However, it is crucial to note that these methods were originally developed for classification, not for inference or estimation purposes. These are distinct goals and involve a distinct trade-off between interpretability and complexity/predictive capacity [78]. Thus, in contrast with what happens in purely predictive settings, studies employing regression/ classification methods to study multivariate sex differences may benefit of using “simple” but highly interpretable models able to provide insight about which brain features contribute and by how much to the multivariate separation of females and males [78]. Additionally, it is important to consider that multivariate differences are inherently larger than univariate ones, and their size grows with the number of variables included [74, 75]. Therefore, multivariate effects and effect sizes are more accurate, meaningful, and interpretable when they summarize a coherent, theoretically justified set of variables rather than when calculated from hundreds or thousands of non-preselected features [74, 75]. Finally, just as it happens with traditional multivariate statistics and effect sizes [68, 69, 74], predictive methods can estimate the size of a difference, but do not provide information about its direction. Thus, it may be beneficial to complement the results obtained from predictive models with those of other methods.

In our study, we employed two distinct and complementary analytical strategies: one based on the classification probabilities obtained from logistic regression models and the other based on projected distances [60]. Both methods allowed us to condense the information of the 18 regions of the SENT_CORE network into a single and continuous metric space, but each of them has particular strengths that address some of the weaknesses of the other and, together, they overcome at least two of the main limitations of classic multivariate methods like MANOVA. Projected distances directly and unambiguously inform about the direction of the multivariate differences ([60] an aspect unaddressed by traditional methods and by those based on classification probabilities). Conversely, logistic regression and other interpretable regression/ classification models help to uncover the structure of the observed multivariate differences (i.e., quantifying the relative contribution of each individual variable to the multivariate effect [79]), an aspect that cannot be addressed by classic multivariate methods and projected distances [68, 69, 74]. Moreover, as these two methods very much differ in their mathematical foundations, producing similar results, would mutually validate their findings.

### Multivariate sex differences and similarities in language-processing brain areas

In both the raw and PCP datasets, individual projected distances and Pclass scores were highly correlated between them (*r* = 0.85 and *r* = 0.84, respectively; see Panels E and F of Fig. 5). This indicates that, regardless their different scale and despite the bounded/ unbounded nature of their outcome variables which resulted in differently shaped distributions in the raw dataset (Panel A of Figs. 4 and 5), the relative position of each individual in the Pclass and projected distances’ continuums were roughly equivalent. Consequently, the estimates of the multivariate sex differences and similarities in the SENT_CORE network calculated from these two distinct outcome variables were quite similar in size, in both the raw and the PCP datasets (see below and Table 3). To our knowledge, this kind of between-method comparisons had not been previously conducted and its results boosts the trustworthiness of the estimates obtained in the present study.

Multivariate sex differences in the SENT_CORE network very much varied depending on whether they were calculated from raw or TIV-adjusted GM_vol_. In the raw dataset, multivariate differences indicated larger raw GM_vol_ in males and were not only “large” accordingly to commonly used benchmarks [51], but also larger than those observed in equivalent univariate analyses and similar in size to those yielded by TIV (the largest macroscopical difference in the brains of females and males; Additional file 1: Table S3A, B). For instance, the Cliff’s delta and Cohen’s U3 values estimated from Pclass scores (0.78 and 96.26%) closely resembled those estimated using the projected distances method (0.66 and 90.74%), which were larger than the averages (0.46 and 80.78%) and that the largest values (0.62 and 92.51%) observed among their univariate counterparts, and became nearly identical to those estimated from TIV (0.78 and 95.58%). On the other hand, multivariate differences in the PCP dataset suggested larger TIV-adjusted GM_vol_ in females and their size was “medium”, surpassing the magnitude of their univariate counterparts. Specifically, the Cliff’s delta and Cohen’s U3 values for Pclass scores (0.36 and 75.5%) and projected distances (0.28 and 68.7%) were quite similar, larger than the average and the maximum values observed in univariate analyses (0.06, 51.02% and 0.16, 59.86%, respectively), but notably smaller than those estimated from TIV.

This study marks the first assessment of multivariate sex differences in GM_VOL_ within the CORE_SENT network. Therefore, our estimates of these differences cannot be directly compared to those from previous studies. Nevertheless, the fact that these estimated differences were larger when calculated from raw than from TIV-adjusted GM_VOL_ aligns with the results of other studies employing classification/ regression methods to assess multivariate sex differences in GM_VOL_ at the whole brain level [31, 40, 77, 80] and specific neural systems [81]. Additionally, the similarity in estimates obtained from two robust methods with different mathematical foundations suggests that these estimates are likely accurate and sound. Consequently, the results of this study contribute to addressing a significant gap in the study of sex differences in the neuroanatomical basis of primary language functions by extending the analysis to the multivariate level. However, these findings should be considered provisional until confirmed by independent studies.

To provide insight and a graphical representation of the structure of the observed multivariate effects identified in Pclass scores, we depicted the nomograms of the fitted LR models (Fig. 6A, B). The inspection of these nomograms (see also Fig. 7D) revealed that, in the raw dataset, only 9 (*T2_3, T2_4, STS_1, STS_2, STS_3, SMG7, INSa3, F3O1,* and *AG2*) out of the 18 brain areas that showed consistent statistically significant differences at the univariate level also reached statistical significance within the multivariate model. Similarly, in the PCP dataset, the two brain areas showing consistent and significant sex differences at the univariate level (*T2_3* and *F2_2*) were joined by three others (*STS1, STS2, T1_4*) in achieving statistical significance as contributors to the multivariate model. This mismatch illustrates that, while the size of the univariate differences in each component of the SENT_CORE network was significantly correlated with their relative contribution within the multivariate model (i.e., the values of the regression coefficients; see Fig. 6C and Additional file 1: Fig. S1), multivariate effects cannot be equated or directly inferred from univariate effects. In other words, to uncover insights at the whole network level, a multivariate approach is required.

On the other hand, the obtained nomograms also demonstrated that, despite the major differences in multivariate effects observed in the raw and PCP datasets, their underlying structure (as indicated by the ordering of the regression coefficients) was quite similar ($\left|rho\right|$= 0.93, Fig. 6D). This unprecedented observation suggests that, although the PCP method removes the influence of TIV-related variation and reduces the size of the estimated multivariate sex differences in GM_vol_, it does not alter the structure of these differences (i.e., the relative contribution of each brain region to the multivariate composites on which the differences are calculated). This became even more evident when examining the nomograms of two additional LR models that, in addition to the volumetric features of the raw and PCP datasets, included TIV as a predictor of the sex categories (Fig. 7A, B). These last two LR models were virtually identical in all aspects, including the ordering of their coefficient values ($\left|rho\right|$>0.99). Furthermore, the ordering of the coefficients observed in all four LR models was highly similar ($\left|rho\right|$> 0.92 in all cases; Fig. 7C), suggesting that these models (and the multivariate sex differences estimated from them) share a similar structure that enables across-models’ comparisons. From these comparisons, it can be concluded that: (1) *T2_3, STS1, STS2* but also *F2_2, T1_4,* and *F3O1* were identified as the local components that more consistently made a statistically significant contribution to the to the multivariate differentiation of the SENT_CORE network in males and females across all models (Fig. 7E); (2) TIV is the brain feature that best separate females and males; (2) once TIV variation is accounted for, the brain features included in the raw and in the PCP datasets contain virtually the same information; and, consequently, (3) the opposite direction and distinct size of the estimated sex differences in those datasets are attributable to the TIV-related variation contained in raw GM_vol_ measurements.

## Limitations

The present study is not without limitations, and it is important to consider at least for four limitations when interpreting our results and valuating our conclusions.

Firstly, as previously mentioned, our study is the first to evaluate the univariate and multivariate sex differences/ similarities in the SENT_CORE network of the SENSAAS atlas, making it challenging to compare our results with those of previous studies. Therefore, our results should be regarded with caution until direct replication studies are conducted.

Secondly, the SENT_CORE is just one of the three networks included in the SENSAAS atlas (the only one specifically constructed as to study language-processing areas and networks in healthy individuals). Consequently, our study does not provide information about the sex differences in all brain areas/networks involved in language processing, but rather focuses on those essential for sentences’ production and comprehension [13].

Thirdly, our sample consisted of right-handed young adults. While this homogenous sample was convenient for isolating the effect of sex, it may limit the generalizability of our results to a broader population. Therefore, readers should interpret our results and conclusions with caution until replicated by independent studies conducted in other samples that differ in potentially relevant variables (e.g., age, scan site, etc.).

Lastly, our study describes anatomical differences/ similarities in a well-validated brain language network but does not allow us drawing conclusions about the causes or potential functional consequences of the observed sex effects in GM_vol_. *Notably, our study did not include any behavioral assessment, thus precluding the evaluation of whether the identified neuroanatomical sex-based differences influence sentence-processing abilities in females and males. Consequently, further studies are needed to investigate these potential functional consequences and their possible relationship with the neuroanatomical and neurofunctional characteristics of the SENT_CORE network*.

## Conclusion: perspectives and significance

In contrast to the conflicting results observed in previous studies investigating potential neuroanatomical sex differences in language-processing areas, the current study confirms the presence of consistent and statistically significant differences in GM_VOL_ between males and females within the SENT_CORE language network, as initially identified by Labache et al. [13]. Additionally, the present study identifies important moderators of these differences. Firstly, these differences are more pronounced when assessed at the entire network level using multivariate methods, as opposed to when examined at the local component level through multiple univariate comparisons. Secondly, the direction and magnitude of these univariate and multivariate differences significantly depend on whether they are calculated from raw GM_VOL_ or GM_VOL_ adjusted for TIV. Specifically, differences appear 'large', indicating larger raw GM_VOL_ in males, but transition to 'small' or 'intermediate' and indicate larger relative volumes in females when adjusting for TIV-related variations. In this context, there is a growing consensus regarding the importance of considering TIV variation as a potential confounding factor that should be statistically controlled for when assessing sex differences in local brain volumes [52,59,82,83]. However, it is worth noting that raw GM_VOL_ and TIV-adjusted GM_VOL_ represent two different types of measures (absolute and relative, respectively), and they seem to address distinct questions rather than providing conflicting answers to the same question. Therefore, we advocate for the evaluation of neuroanatomical sex differences using both raw and TIV-adjusted measures whenever possible, or at the very least, when (as in the case of the present study) it is unclear which of these two measurements, if any, may be relevant in explaining cognitive processes or other behavioral phenomena.

### Supplementary Information

**Additional file 1.** Additional tables and figures.

## Data Availability

This study was primarily conducted using data from the open source 1200 Subject Release (S1200) of the Human Connectome Project (HCP). The access to this sample should be directly requested to the Washington University—University of Minnesota Consortium of the Human Connectome Project (WU-Minn HCP).

## References

[1] Halpern DF. Sex differences in cognitive abilities, 4th edn. 2013.

[2] Wallentin M (2009). Putative sex differences in verbal abilities and language cortex: a critical review. Brain Lang.

[3] Marini A (2023). The beauty of diversity in cognitive neuroscience: The case of sex-related effects in language production networks. J Neurosci Res..

[4] Kimura D (2004). Human sex differences in cognition, fact, not predicament. Sex Evol Gend..

[5] Wallentin M (2020). Gender differences in language are small but matter for disorders. Handb Clin Neurol..

[6] Chilosi AM, Brovedani P, Cipriani P, Casalini C (2023). Sex differences in early language delay and in developmental language disorder. J Neurosci Res..

[7] Thompson T, Caruso M, Ellerbeck K (2003). Sex matters in autism and other developmental disabilities. J Learn Disabil..

[8] Sato M (2020). The neurobiology of sex differences during language processing in healthy adults: a systematic review and a meta-analysis. Neuropsychologia.

[9] Kaiser A, Haller S, Schmitz S, Nitsch C (2009). On sex/gender related similarities and differences in fMRI language research. Brain Res Rev.

[10] Blanton RE, Levitt JG, Peterson JR, Fadale D, Sporty ML, Lee M (2004). Gender differences in the left inferior frontal gyrus in normal children. Neuroimage.

[11] Wilke M, Holland SK, Krägeloh-Mann I (2007). Global, regional, and local development of gray and white matter volume in normal children. Exp Brain Res.

[12] Etchell A, Adhikari A, Weinberg LS, Choo AL, Garnett EO, Chow HM (2018). A systematic literature review of sex differences in childhood language and brain development. Neuropsychologia.

[13] Labache L, Joliot M, Saracco J, Jobard G, Hesling I, Zago L, et al. A SENtence Supramodal Areas AtlaS (SENSAAS) based on multiple task-induced activation mapping and graph analysis of intrinsic connectivity in 144 healthy right-handers. 2019;224:859–82. 10.1007/s00429-018-1810-2 10.1007/s00429-018-1810-2 PMC6420474 30535758

[14] Tremblay P, Dick AS (2016). Broca and Wernicke are dead, or moving past the classic model of language neurobiology. Brain Lang.

[15] Ullman MT, Miranda RA, Travers ML (2007). Sex differences in the neurocognition of language. Sex Differ Brain From Genes to Behav..

[16] Vigneau M, Beaucousin V, Hervé PY, Jobard G, Petit L, Crivello F (2011). What is right-hemisphere contribution to phonological, lexico-semantic, and sentence processing? Insights from a meta-analysis. Neuroimage.

[17] Sidtis JJ (2007). Some problems for representations of brain organization based on activation in functional imaging. Brain Lang.

[18] Sommer IE, Aleman A, Somers M, Boks MP, Kahn RS (2008). Sex differences in handedness, asymmetry of the Planum Temporale and functional language lateralization. Brain Res.

[19] Amunts K, Zilles K (2012). Architecture and organizational principles of Broca’s region. Trends Cogn Sci..

[20] Price CJ (2012). A review and synthesis of the first 20years of PET and fMRI studies of heard speech, spoken language and reading. Neuroimage.

[21] Harrington GS, Farias ST (2008). Sex differences in language processing: functional MRI methodological considerations. J Magn Reson Imaging..

[22] Button KS, Ioannidis JPA, Mokrysz C, Nosek BA, Flint J, Robinson ESJ (2013). Power failure: Why small sample size undermines the reliability of neuroscience. Nat Rev Neurosci..

[23] Ioannidis JPA (2005). Why most published research findings are false. PLoS Med..

[24] Kroliczak G, Gonzalez CL, Carey DP (2019). Editorial: manual skills, handedness, and the organization of language in the brain. Front Psychol.

[25] Romeo RR, Leonard JA, Robinson ST, West MR, Mackey AP, Rowe ML (2018). Beyond the 30-million-word gap: children’s conversational exposure is associated with language-related brain function. Psychol Sci.

[26] Brito NH, Noble KG (2018). The independent and interacting effects of socioeconomic status and dual-language use on brain structure and cognition. Dev Sci.

[27] Schlaepfer TE, Harris GJ, Tien AY, Peng L, Lee S, Pearlson GD (1995). Structural differences in the cerebral cortex of healthy female and male subjects: a magnetic resonance imaging study. Psychiatry Res Neuroimaging..

[28] Luders E, Narr KL, Zaidel E, Thompson PM, Toga AW (2006). Gender effects on callosal thickness in scaled and unscaled space. NeuroReport.

[29] Harasty J, Double KL, Halliday GM, Kril JJ, McRitchie DA (1997). Language-associated cortical regions are proportionally larger in the female brain. Arch Neurol.

[30] Sanchis-Segura C, Ibañez-Gual MV, Adrián-Ventura J, Aguirre N, Gómez-Cruz ÁJ, Avila C (2019). Sex differences in gray matter volume: How many and how large are they really?. Biol Sex Differ.

[31] Sanchis-Segura C, Ibañez-Gual MV, Aguirre N, Gómez-Cruz ÁJ, Forn C (2020). Effects of different intracranial volume correction methods on univariate sex differences in grey matter volume and multivariate sex prediction. Sci Rep.

[32] Dhamala E, Ooi LQR, Chen J, Kong R, Anderson KM, Chin R (2022). Proportional intracranial volume correction differentially biases behavioral predictions across neuroanatomical features, sexes, and development. Neuroimage.

[33] Hu M, Lou Y, Zhu C, Chen J, Liu S, Liang Y (2023). Evaluating the impact of intracranial volume correction approaches on the quantification of intracranial structures in MRI: a systematic analysis. J Magn Reson Imaging.

[34] Liu D, Johnson HJ, Long JD, Magnotta VA, Paulsen JS (2014). The power-proportion method for intracranial volume correction in volumetric imaging analysis. Front Neurosci..

[35] Vigneau M, Beaucousin V, Hervé PY, Duffau H, Crivello F, Houdé O (2006). Meta-analyzing left hemisphere language areas: phonology, semantics, and sentence processing. Neuroimage.

[36] Dronkers NF, Wilkins DP, Van Valin RD, Redfern BB, Jaeger JJ (2004). Lesion analysis of the brain areas involved in language comprehension. Cognition.

[37] Wilcox RR. Introduction to robust estimation and hypothesis testing. Introduction to robust estimation and hypothesis testing, 2nd edn; 2022.

[38] Van Essen DC, Smith SM, Barch DM, Behrens TEJ, Yacoub E, Ugurbil K (2013). The WU-minn human connectome project: an overview. Neuroimage.

[39] Kuhn M. caret: Classification and Regression Training [Internet]. 2022. https://cran.r-project.org/package=caret

[40] Sanchis-Segura C, Aguirre N, Cruz-Gómez ÁJ, Félix S, Forn C (2022). Beyond, “sex prediction”: estimating and interpreting multivariate sex differences and similarities in the brain. Neuroimage.

[41] Hastie T, Tibshirani R, Friedman J. The elements of statistical learning, data mining, inference, and prediction, Second Edition. Springer; 2009.

[42] Ali A, Shamsuddin SM, Ralescu AL (2015). Classification with class imbalance problem: a review. Int J Adv Soft Comput Appl..

[43] Leys C, Ley C, Klein O, Bernard P, Licata L (2013). Detecting outliers: do not use standard deviation around the mean, use absolute deviation around the median. J Exp Soc Psychol.

[44] R Core Team. R: A language and environment for statistical computing. R Foundation for Statistical Computing, [Internet]. Viena (Austria); 2020. https://www.r-project.org/.

[45] Benjamini Y, Hochberg Y (2018). Controlling the false discovery rate: a practical and powerful approach to multiple testing. J R Stat Soc Ser B.

[46] Pastore M, Calcagnì A (2019). Measuring distribution similarities between samples: a distribution-free overlapping index. Front Psychol.

[47] Pastore M (2018). Overlapping: a R package for estimating overlapping in empirical distributions. J Open Source Softw.

[48] Cohen J (1988). Statistical power analysis for the behavioral sciences.

[49] Grissom RJ, Kim JJ (2012). Effect sizes for research: univariate and multivariate applications. Multivariate application tests.

[50] Cliff N (1993). Dominance statistics: ordinal analyses to answer ordinal questions. Psychol Bull.

[51] Mangiafico S. Rcompanion: functions to support extension education program evaluation. R package version 2.2.2. 2019.

[52] Grice JW, Barrett PT (2014). A note on Cohen’s overlapping proportions of normal distributions. Psychol Rep.

[53] Davison A., Hinkley DV. Bootstrap Methods and Their Applications [Internet]. Cambridge University press; 1997. http://statwww.epfl.ch/davison/BMA/

[54] Barnes J, Ridgway GR, Bartlett J, Henley SMD, Lehmann M, Hobbs N (2010). Head size, age and gender adjustment in MRI studies: A necessary nuisance?. Neuroimage.

[55] Pintzka CWS, Hansen TI, Evensmoen HR, Håberg AK (2015). Marked effects of intracranial volume correction methods on sex differences in neuroanatomical structures: a HUNT MRI study. Front Neurosci..

[56] Lippa R, Connelly S (1990). Gender diagnosticity: a new Bayesian approach to gender-related individual differences. J Pers Soc Psychol.

[57] Harrell FE. rms: Regression modeling strategies. [Internet]. 2022. https://hbiostat.org/R/rms/

[58] Wilcox RR, Rousselet GA (2018). A guide to robust statistical methods in neuroscience. Curr Protoc Neurosci..

[59] Cohen P, Cohen J, Aiken LS, West SG (1999). The problem of units and the circumstance for POMP. Multivariate Behav Res..

[60] Wilcox RR (2004). A multivariate projection-type analogue of the Wilcoxon—Mann—Whitney test. Br J Math Stat Psychol.

[61] Wilcox RR, Muska J (1999). Measuring effect size: a non-parametric analogue of ω2. Br J Math Stat Psychol.

[62] Ritchie SJ, Cox SR, Shen X, Lombardo MV, Reus LM, Alloza C (2018). Sex differences in the adult human brain: evidence from 5216 UK biobank participants. Cereb Cortex.

[63] Williams CM, Peyre H, Toro R, Ramus F (2021). Neuroanatomical norms in the UK Biobank: the impact of allometric scaling, sex, and age. Hum Brain Mapp.

[64] Brun CC, Leporé N, Luders E, Chou YY, Madsen SK, Toga AW (2009). Sex differences in brain structure in auditory and cingulate regions. NeuroReport.

[65] Luders E, Narr KL, Thompson PM, Woods RP, Rex DE, Jancke L (2005). Mapping cortical gray matter in the young adult brain: effects of gender. Neuroimage.

[66] Good CD, Johnsrude I, Ashburner J, Henson RNA, Friston KJ, Frackowiak RSJ (2001). Cerebral asymmetry and the effects of sex and handedness on brain structure: A voxel-based morphometric analysis of 465 normal adult human brains. Neuroimage.

[67] Kurth F, Jancke L, Luders E (2017). Sexual dimorphism of Broca’s region: More gray matter in female brains in Brodmann areas 44 and 45. J Neurosci Res..

[68] Huang FL (2020). MANOVA: a procedure whose time has passed?. Gift Child Q.

[69] Bathke AC, Friedrich S, Pauly M, Konietschke F, Staffen W, Strobl N (2018). Testing mean differences among groups: multivariate and repeated measures analysis with minimal assumptions. Multivariate Behav Res..

[70] Bzdok D. Classical statistics and statistical learning in imaging neuroscience [Internet]. Front. Neurosci. Frontiers Media S.A.; 2017. p. 543. www.frontiersin.org. Accessed 13 May 2021. 10.3389/fnins.2017.00543 PMC5635056 29056896

[71] Mišić B, Sporns O (2016). From regions to connections and networks: New bridges between brain and behavior. Curr Opin Neurobiol..

[72] O’Toole AJ, Jiang F, Abdi H, Pénard N, Dunlop JP, Parent MA (2007). Theoretical, statistical, and practical perspectives on pattern-based classification approaches to the analysis of functional neuroimaging data. J Cogn Neurosci..

[73] Davatzikos C (2004). Why voxel-based morphometric analysis should be used with great caution when characterizing group differences. Neuroimage.

[74] Del Giudice M. Measuring sex differences and similarities. In: VanderLaan, D.P.; Wong WI, editor. Gender and sexuality development. Contemporary theory and research, 1st ed. New York; 2019.

[75] Eagly AH, Revelle W (2022). Understanding the magnitude of psychological differences between women and men requires seeing the forest and the trees. Perspect Psychol Sci..

[76] Kim K, Joo YY, Ahn G, Wang HH, Moon SY, Kim H (2022). The sexual brain, genes, and cognition: a machine-predicted brain sex score explains individual differences in cognitive intelligence and genetic influence in young children. Hum Brain Mapp.

[77] van Eijk L, Zhu D, Couvy-Duchesne B, Strike L, Lee A, Hansell N, et al. Are sex differences in human brain structure associated with sex differences in behaviour? Psychol Sci [Internet]. PsyArXiv; 2021. https://psyarxiv.com/8fcve/. Accessed 2 May 2021. 10.1177/0956797621996664 PMC8726594 34323639

[78] Bzdok D, Ioannidis JPA (2019). Exploration, inference, and prediction in neuroscience and biomedicine. Trends Neurosci..

[79] Bzdok D, Engemann D, Thirion B (2020). Inference and prediction diverge in biomedicine. Patterns.

[80] Wiersch L, Hamdan S, Hoffstaedter F, Votinov M, Habel U, Clemens B (2022). Accurate sex prediction of cisgender and transgender individuals without brain size bias. BioRxiv..

[81] Matte Bon G, Kraft D, Comasco E, Derntl B, Kaufmann T (2023). Modeling brain sex in the limbic system as phenotype for female-prevalent mental disorders. MedRxiv..

